# A Three Layered Decentralized IoT Biometric Architecture for City Lockdown During COVID-19 Outbreak

**DOI:** 10.1109/ACCESS.2020.3021983

**Published:** 2020-09-04

**Authors:** Manjur Kolhar, Fadi Al-Turjman, Abdalla Alameen, Mosleh M Abualhaj

**Affiliations:** 1 Department of Computer SciencePrince Sattam Bin Abdulaziz University 204568 Wadi Ad-Dawasir 11990 Saudi Arabia; 2 Research Center for AI and IoTArtificial Intelligence DepartmentNear East University 52988 99138 Mersin Turkey; 3 Department of Networks and Information SecurityAl-Ahliyya Amman University 74429 Amman 19328 Jordan

**Keywords:** Face detection, cascaded convolution neural network, edge computing, cloud computing, IoT

## Abstract

In this article, we have built a prototype of a decentralized IoT based biometric face detection framework for cities that are under lockdown during COVID-19 outbreaks. To impose restrictions on public movements, we have utilized face detection using three-layered edge computing architecture. We have built a deep learning framework of multi-task cascading to recognize the face. For the face detection proposal we have compared with the state of the art methods on various benchmarking dataset such as FDDB and WIDER FACE. Furthermore, we have also conducted various experiments on latency and face detection load on three-layer and cloud computing architectures. It shows that our proposal has an edge over cloud computing architecture.

## I. Introduction

It is reported that by 2020, more than 50 billion devices will be connected through radio communications. IoT network and its physical devices such as sensors, mobile devices, radio identification tag, and actuators are programmed to collect data from the user environment. IoT network, are employed in many fields including security, agriculture, utility management, smart homes and health care systems [1]–[6]. These capabilities are further extended to IoT enabled services with smart devices. Such combinations of services are more suitable to users. Because they have certain features which makes more competitive among other wireless devices, and these features are long range communications, low data rate, consumption of less energy and less cost. Smartphone enabled IoT services have become future networking paradigm and service framework in which distributed services and physical object will be arrayed together for useful information that enables intelligent services. Biometric services have been studied and implemented for a particular services, however, such settings require privacy and security embedded in them to have controlled environment. The surveillance such as face detection in residential area greatly required specially during pandemic with high risk of community spread of contagious disease within a community. Such enriched technologies in small sized embedded physical devices enabled the growth of more advanced computing devices that are equipped with enough computational power to analyze and recognize face on their own without the intervention of Cloud computing architecture. IoT and overlaying technologies that have reached flawless level of management of devices and communication protocol into the level of perfection. The combination of these two technologies made it possible to produce three layered edge computing. Fog computing is expansion of cloud computing which allows building an infrastructure to allocate the workload to balance between two or more Cloud entities.

During the pandemic, the population will be under lockdown and restrictions on the movement of people. To impose such lockdown to curb the pandemic with the help of technology especially IoT and physical smart devices are necessary because the virus can spread easily if we use human power to control residential areas.

The objective of this work is to develop an IoT the biometric framework which can detect and recognition of face using three-layered edge architecture. To this end, we have utilized the capacity of Multi-task Cascaded Convolutional Network architecture (MCCNN), efficiently using Raspberry Pi, for robust detection and recognition of a face. The proposed network compared with the state-of-the-art method on various datasets of challenging benchmarks, which further strengthens the proposed method to avoid the training framework from scratch. In the proposed MCCNN network, only a few features across multiple channels are used, while advanced layers for pre-training remained intact. In the proposed system, we have ported Computer Vision library to the Raspberry Pi computer. This setting allowed us to surveil people’s movement necessary tools under lockdown condition. Secondly, we have implemented face detection methods on edge computing architecture using three layered architecture namely edge layer, application layer, and physical layer, and the required configuration items are installed and executed on Raspberry Pi computers. The remainder of this article is structured as follows: Division II reviews the existing state of the art methods, image processing frameworks for handling biometric data. Division III introduces the main concepts of our proposed framework. It also describes the developed three-layered architecture for processing the face data in the residential areas and at any time. Division IV describes the various experimental scenario used to validate the proposed architecture. The main outcomes of the proposed architecture are discussed. Section V concludes our research and discusses further research.

## II. Related Work

During the COVID-19 pandemic outbreak, it becomes necessary for the general public to follow steps as advocated by the city councilors to contain COVID-19. There are many rules and guidelines suggested across the world, such as partially lifting of lockdown, complete lockdown, and allowing few family members to access general and essential goods or city council will take its own course of action to supply essential goods. To follow such rules and keeping an eye on each and every person becomes difficult and cumbersome. So, taking advantage of IoT based solutions becomes the norm. Hence, in this article, we have built an IoT based biometric solution to help people to follow guidelines under lockdown conditions.

IoT based technology has been timely surveyed [27]–[29]. Authors have presented a survey of IoT based technology, their security features, embedded hardware design, and competencies of their networking stack have also studied. Furthermore, IoT based required essential technologies for the IoT service infrastructure, existing trends, and their unique features. IoT based new technologies are also introduced with the help of 3GPP radio technology which incurred low power consumption and cost.

There are two regions specifically used in the face recognition and detection framework [7]–[13], which are named as complete face region and occlusion verification. Most of these proposed algorithms are rare to verify occlusion and hence occlusion is still at large. However, there are a number of researchers have tried to solve the face occlusion by using filter functions [23]. However, various temporal information along with the skin have become important features for occlusions problems [24]. Furthermore, some researchers have discovered more features in parts to recognize [25], [26]. Most of these methods have proved to handle occlusions from different regions of the human body, however, they failed to handle occlusion under severe conditions. Although, a face-based application using IoT based on various methods such as color, contour, matching based methods have contributed to face detection and recognition research area. These methods have a computational requirement when face is covered.

Face detection is one of the widely studied problems in image processing. The authors’ Viola and Jones have proposed the two basic image processing algorithms on face detection using boosted cascade structure and simple features. Till now, these algorithms are being used in many applications ranging from industry and academia. However, these algorithms are suitable for normal conditions but not a good candidate for harsh conditions such as low light and occlusion of expression. These two algorithms are built using the Harr technique, and it is proven to be insufficient when faces have variations.

In [13], boosting was introduced with the AdaBoost algorithm, the AdaBoost algorithm combines classifiers to bring more accuracy to the classifiers. It is an ensemble method and works iteratively. AdaBoost combines poorly executing classifiers to form strong classifiers so as to produce high accuracy. Lately, it proved that AdaBoost won’t work in multiclass cases. However, there were efforts rendered to enhance AdaBoost for multi-class [14]. Most recent research is also based on AdaBoost, however, but they are utilized facial expressions in the behavioral analysis of emotions, cognitive science, and social interactions [15], [16].

Authors [15], have proposed AdaBoost with dynamic time wrapping for facial expression recognition on boosted features. The features of the face are collected from the collection of facial expression images based on tracking facial landmarks.

In [16], they have used Viola Jones, histograms of oriented gradients, and multi-class SVM. Authors have used SVM (support vector machine) multiclass methods to train data from 5 different angle image captured from CCTV positioned from the walk through the metal detector. Authors [17], have introduces a technique using deep learning to detect a face. They have applied a convolutional neural network model along with AdaBoost.

In [18], the authors presented facial expression recognition model, it detects the emotional states in real time. Further, it allows the host machine to interact with the user and identifies the frontal face along with the region of interest (ROI), collects features from suitable facial landmarks, and classifies the facial expressions. In [19], authors have proposed a model for breeding classifier ensembles based on clustering. However, a continuous stream of video data makes the complete system burdened large memory. Hence, they have built a framework to collects only the informative data from the memory by applying the clustering technique. Authors [20], have suggested Haar techniques to protect images apart from image detection. They have utilized the cloud resources for collecting Haar feature from privacy-preserved images from the cloud.

In [21], authors have used image processing and execution for handheld devices particularly for sports and entertainment applications. They claimed that complex dependencies of service modules aimed at optimizing the execution time and energy consumption during real-time execution of services.

Authors of [22], have designed a face occlusion technique using an IoT device particularly focused on criminal behavior. Furthermore, it uses deep learning techniques and utilizes gradient and shape cues to yield the best results in face detection using face occlusion.

Nowadays, face detection can be used for a variety of applications apart from just for access control or identification. Facial biometrics has also been studied for demographic information, for example, age, gender, and mood. Such parameters can be fed to the Interactive billboard or sign module for determining reactions. Hence, face detection is not only broadened its scope in terms of application apart from its core issues related to image processing. However, its core issues are still being researched such as time spent in processing is still a challenge, and face occlusions because of various reasons related to the face captured and the surrounding atmosphere. These problems are still at large and the research fraternity to suggest a more efficient solution to avoid the aforementioned issues with respect to image processing and identification.

## III. A Three Layered Decentralized IoT Biometric Architecture

**FIGURE 1.**
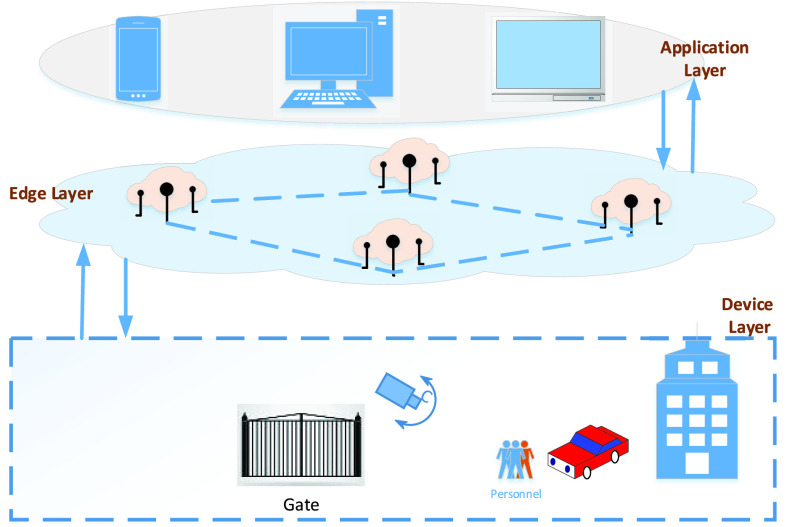
Proposed architecture, consists of a device layer, an edge layer and an application layer.

To have scalability and management of the complete proposed system we have proposed edge-based monitoring and control system for the residents. Hence, the core of the proposed system is edge layer, because it receives important data and it controls the camera other sensing devices. Secondly, we have an application layer that uses smart devices to interact with the edge layer to have data from the sensors and cameras. Device layer implements, core proposal of face detection and recognition to restrict residents. Residents, are required to be seized in their respective homes, however, to stay during the lockdown is difficult and sometimes lockdown is partially relaxed for few hours a week so that people can visit their shopping centers for buying essential goods. For such situations, allowing only one person from each family is accepted to go out for shopping. To enforce such laws, we have designed image processing-based solution. Figure 1 shows the architecture used in this research work. The mapping between the application layer, device layer, and edge layer is a straight forward communication. The lowest layer is called the device layer, it is collection of physical devices connected together to achieve certain work assigned to them by application layer through edge layer. The edge layer is the backbone of the complete system which sits between the physical layer and the application layer and has computational units. The application layer, it is completely controlled by the human for their intended jobs, in our case, we have face identification and recognition system.

### A. Physical Layer

It hosts several devices, and their job is to constantly collect face image data from the residential area especially at the entrance. These devices can be fixed cameras and sensor smart devices. Each of these devices has ported on Raspberry Pi device and hence they execute numerous jobs on their own. It contains capture and face detection and database connection modules. The capture module captures the face of the person who intends to leave the apartment. This face image will be sent to the cloud-based application server and, once the face is detected, the person’s information is updated.

**FIGURE 2.**
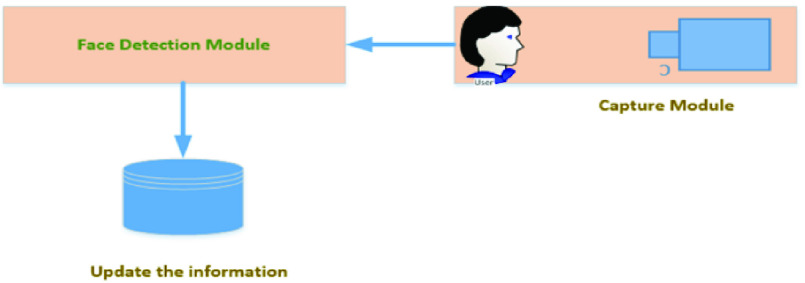
Physical Layer communication, its capture module.

Fig 2 physical layer, contains a camera to capture the face, and it sends captured images to the cloud-based database and application server. The application server recognizes the face and blocks other family members. The database of images is gathered from each residential area, presently we have used a set of images from (WIDER FACE dataset) This database also hosts house wise or flatwise (house) images of the residents. Presently, our proposal is in the initial stage of development, so it hosts very few images of 100 flats data, but it can be employed to run into a real environment.

### B. The Edge Layer

In this section, we brief the design of the edge layer and its working. Mainly, the edge layer receives and stores information in database originating from physical layer for aiding in decision making (see algorithm 1) to recognize and to transfer information to others. The device layer receives data from the camera and sensors and the data is dispatched inside the device layer for further analyzing purpose. The Raspberry Pi devices nodes allow a quick response time and without compromising the computation of the system. The main job of the edge layer is to combine and compute data and from various kinds of devices. Hence, gathered information from different IoT devices to have a better understanding and interpretation of the ground data and ensure the best decision.

Algorithm 1 **Algorithm for Detection of Face, Using Multi-Task Cascaded Convolutional Network**
Input: Capture the Image
Output: Confidence of the bounding boxes and facial landmarks.
1: Capture the image of the person
2: Pass it to daemon process to process on the image
3: Create pyramid of image; send this to stage 1
4: Develop bounding boxes for each image; from top left corner of the image to down right
5: Delete all the bounding boxes who less confidence
6: Induce NMS method, for each scaled image
7: Readjust the bounding boxes
8: Padding
9: Feed output to the second stage
10: Again, delete the bounding boxes
11 Apply NMS methods to all bounding boxes with lower confidence level
12: Re-adjust the bounding boxes
13 Padding
14: Feed output to the third stage
15: Delete unnecessary bounding boxes
16: Convert high confidence bounding into facial landmarks
17: Result: Confidence of the boundin boxes and facial landmarks

**FIGURE 3.**
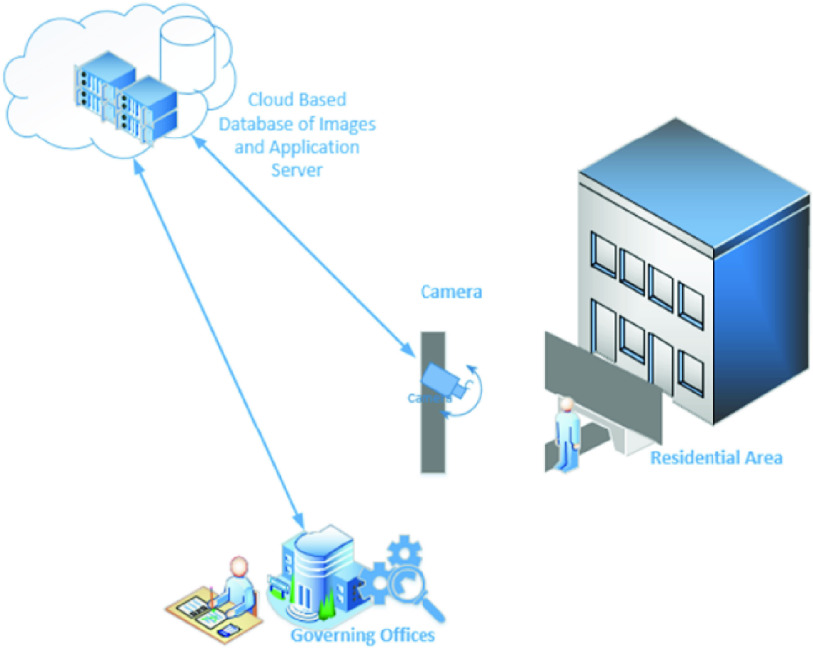
Proposed Method, edge layer with application server and database interacting with cloud service provider.

The edge layer, Fig 3, is consists of cloud architecture and hosts application server and cloud database, which is continuously receives data from physical layer devices. Every time a physical layer device captures face image and reported to the edge layer in order to recognize and ultimately take suitable countermeasures.

The application server takes care of the job of bookkeeping of residents who are out & in and block people who are not eligible to leave houses. This application is connected to the local authorities to have a watch on the residents. Through local authorities’ police can be alerted to find mischiefs people who break laws of lockdown.

With respect to the application server, which executes major modules called deep learning module built using CNN along with a multi-task cascaded method. It has three modules, namely CNN based series of bounding boxing of the captured face image, secondly, regression of bounding boxes, and image enhancement. Bounding boxes created by the previously yield multiple bounding boxes. Therefore, to avoid overlapping of such bounding boxes, non-maximum suppression (NMS) is adopted to refine the bounding boxes given by detectors. All these images are fed to another Refiner network, which is the second module of the project. This second module is applied to further enhance the image by using bounding box regression. The third module is further enhancing the image by classifying main features such as the eye (left and right), nose, and mouth (right and left) of the face.

### C. Face Detection Using CNN Architecture

Algorithm 1, shows the steps used to detect face using library C++. It is proven that multiple CNN to detect faces have complications because of the fact that lack of multiplicity of weights that have resulted in the production of discriminative descriptions. Hence, face detection needs a smaller number of filters and a greater number of discriminative descriptions. We have used the python library to develop Face Detection with Deep Learning. Our proposed method is available in the form of a programming library called OpenCV library, it is written in the C++ computer vision library which provides a python interface. Hence, pre-trained faces are also employed with this library and it comes with plugins to train any model of your own dataset. Our proposed method has three stages as follows;

The proposed method places the camera at the front gate that captures the face of the person and produces different sized images of the face, which looks like image pyramid. These images are fed to the Multi-task Cascaded Convolutional Network (MTCNN) daemon process, which recognizes the face eventually produces five facial landmarks’ positions. In the first stage, the kernel passes through the face image from top left corner browses (technically it is called stride by 2 pixels) till it gets complete landmarks of face coordinates by bounding boxes. Shifting of two cells which is called stride, helps the normal central processing unit to perform a calculation without any bottleneck. The key elements of the first stage, weights and biased are trained so that they can accurately produce bounding boxes for the captured image of size $12\times 12$ kernel. Hence, it keeps on generating bounding boxes with certain confidence levels, the daemon process deletes the bounding boxes with fewer confidence levels and considers only the highest confidence levels. However, this process will produce a lot of boxes which overlap each other, finally, the daemon process uses the NMS process, which reduce the number of bounding boxes and produces a list of the highest confidence level of boxes, and then the information will be fed to the second stage.

In the second stage, each bounding box has an array of pixels created to form a new array. For example, if the bounding box is slightly away from the portion of the image then we consider only the pixel values which are inside the bounding box and mark the rest of the pixels as zero, this processing called padding. The processing of padding for a processed image enables a more accurate analysis of the image. When we continue to perform padding, our proposed method removes lower confident boxes, and employ suppression techniques on lower confident boxes to eliminate them. Then newly formed bounding boxes are built using the P-Net bounding boxes; hence they need conversion to form standard coordinates. The output of the second stage is quite similar to the first stage, however, it contains the coordinates of the new face image, more truthful bounding boxes, for each bounding box.

In the final stage, it starts with padding of bounding boxes who are out of bounds and resize the pixels. This final process yields the coordinates bounding box, facial landmarks, and the confidence level of each bounding box. To get all this, by removing the bounding boxes of lower confidence and standardize the coordinate of bounding boxes and facial landmarks by using the NMS method.

Once the image is received from the camera will be sent to the application server. The application server runs through 17 steps to produce facial landmarks. Apart from running the daemon process, it also handles updating the image-based information in the database and it also helps local governing bodies to look after any miscreants who breaks the law of lockdown.

### D. Bounding Boxes

In image detection, we have applied bounding boxes to find the facial landmarks. The bounding box is a box that is identified with the help of coordinates in the upper-left corner and lower-right corner of the box. In each stage, we have applied these bounding boxes to find an image with the highest level of confidence. In this research, we have used regression of predicted bounding boxes and ground truth boxes are computed in each stage with the help of cross-entropy loss.

\begin{equation*} L_{i}^{det}=-\left ({y_{i}^{det}log\left ({p_{i} }\right)+(1-y_{i}^{det})(1-log(p_{i})) }\right)\tag{1}\end{equation*}

The aforementioned Eq. 1 is derived from the Kaipeng Zhang et.all. Eq 1 produces face with the ground-truth label. Eq 2, gives the bounding boxes derived from the Euclidean loss for each pixel. It contains regression target denoted as against the ground truth. Each of the stages is run through bounding boxes regressions, this results in multiple edge detection of the face. Hence, to reduce the bounding boxes with lower confidence NMS is applied.

**FIGURE 4.**
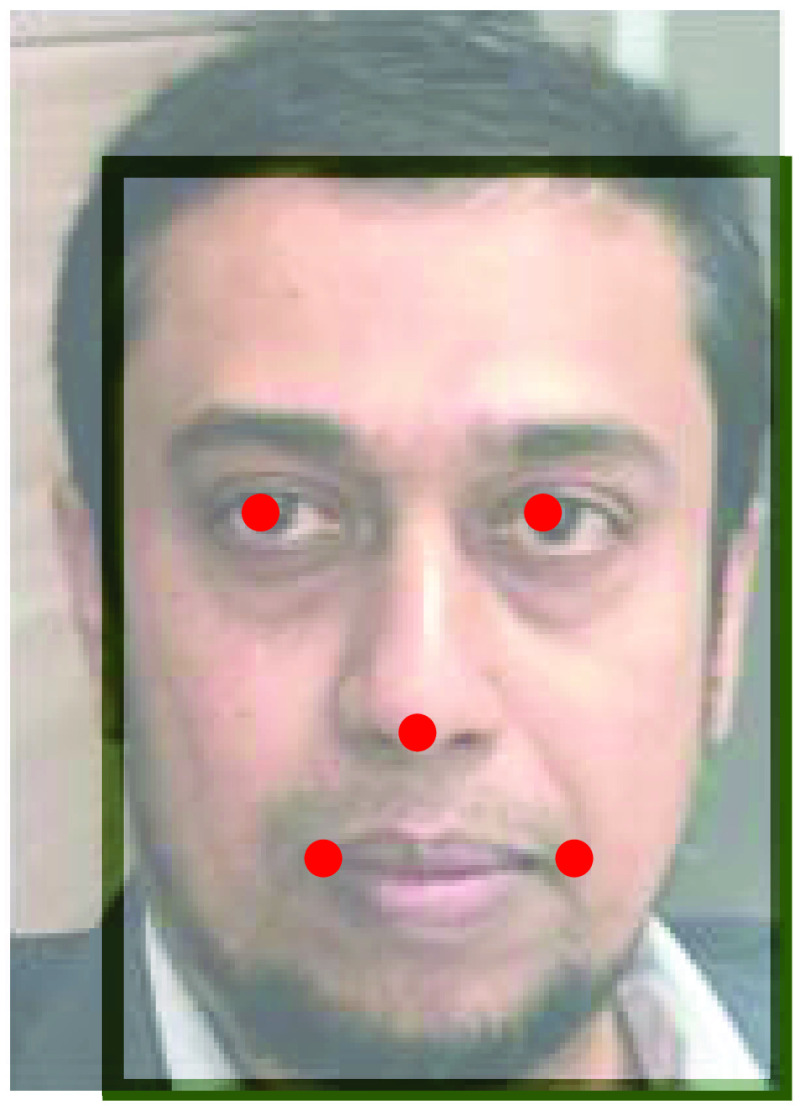
Facial landmark localization using MTCNN method on any given dataset.

Fig 4. Shows. Facial landmarks, we can also apply facial landmark detection can also be calculated by Euclidean loss [32].

\begin{equation*} L_{i}^{Landmark}=\left \|{ \hat {y}i^{Landmark}-\acute {y}i^{Landmark} }\right \|\tag{2}\end{equation*}

Eq. 2 consists of facial coordinate and ground truth coordinates. This equation yields us facial landmarks when it is bounding box regressively applied on the face image by using all three phases (P-net, R-Net, and O-Net). Hence, masked persons cannot be identified.

To conveniently describe abbreviations, and corresponding definitions used throughout manuscript are listed in Tab. 1

\begin{equation*} L_{\textrm {i}}^{\textrm {box}} =\left \|{ {y_{i}^{box} -y\left.{ {_{i}^{box}} }\right \|} }\right.\tag{3}\end{equation*}

Our proposed method returns similarity index as we input two same person’s images, whereas it produces lower similarity index when the face recognition receives a different person images. Hence, it should be trained to achieve the aforementioned capabilities, therefore following are the steps are used to detect face and comparison purpose.

• Detect/identify by using a face detection model
• Predict landmarks for the image identified in step 1
• Using these landmarks calculate face encodings.
• Compare the face encodings of known images with those from saved database images to identify the face.

**FIGURE 5.**
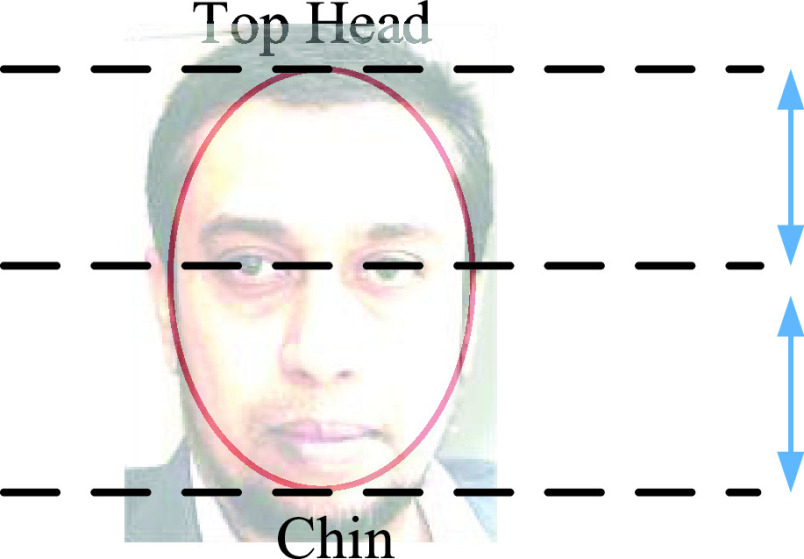
Guidelines for drawing ellipses around face regions from FDDB.

To have uniformity across all the dataset of multiple face annotators, we used a set of instructions as suggested in our datasets. However, the FDDD dataset uses ellipse to fit facial landmarks as shown in Fig 5.

**TABLE 1:** Abbreviations

Symbol	Description
$L_{i}^{det}$	Entropy loss function
$y_{i}^{det}$	Ground truth
$L_{{i}}^{\mathrm{box}} $	Bounding box
$W$	Waiting Time
$T_{\delta }$	Average transmission
$P_{Q}$	Probability of the Queue
$\mu $	Service rate
$\rho $	Utilization factor
$L_{C} $	Cloud utilization
$UD$	UE upload and download line
$L_{e}$	Delay at the edge

### E. Edge Computing

Edge computing is directly communicating with the physical layer environment node or sensors, camera, and it is collectively called IoT. An IoT is represented as 4 tuples ${ < C}_{t},L_{g},F_{a},N_{L}>$, where $C_{t}$ is the computational time spent on the task. The performance of edge computing depends on $C_{t}$ because IoT consumes more computational time then the performance of the complete framework will be degraded and the throughput of the system will also be further degraded, and it will not be a useful candidate for a real-time decision-making application. The second tuple ${L}_{g}$, is geolocation of the IoT in our case it is either camera or thermal sensors. $F_{a}$, represents the availability of the IoT device and its computational load will be shared among the available IoT’s if it is not available. Finally, $N_{L}$ represents the latency of the particular geo-located IoT.

Furthermore, the mean arrival time of the request for face recognition requests can be distributed within a residential area is given by the M/M/c and queueing delay can be had from the following. An M/M/c is a queueing system with impatient residence and synchronous lockdown policy. Residence arriving according to a Poisson process rate. The service of capturing face image by the small computer node who sever residence on a first come first basis and time spent on each residence is exponentially distributed $\frac {1}{\mu }$. When a node finds none of the residences in the queue then all nodes who are serving will consume zero bandwidth otherwise, they will serve residents who are willing to leave the campus. The time spent idle is exponentially distributed with a mean $\frac {1}{\gamma }$. Once the node finds faces then all node who are idle will be active and this also exponentially distributed with mean $\frac {1}{\xi }$. The delay incurred due to this variation in the service and edge cloud computing which resembles the data center. In edge computing, the arrival of the task is processed by nodes hence, communication in the edge computing increases when the load rises and jobs (residents) are queued till their face is processed.

\begin{align*} \text {Delay}=&d_{IoT}=W+\frac {1}{\mu }+T_{\delta } \\=&P_{Q}\ast \frac {\rho }{\eta \left ({1-\rho }\right)}+\frac {1}{\mu }+T_{\delta } \tag{4}\\ \text {Probability of the Queue}=&P_{Q}=\frac {\left ({c\rho }\right)^{c}}{c!}\frac {1}{1-\rho }\rho _{0} \tag{5}\\ \text {Initial Probability}=&\left [{ \sum \limits _{k=0}^{c} \frac {\left ({c\rho }\right)^{c}}{k!} +\frac {\left ({c\rho }\right)^{c}}{c!}\times \frac {1}{1-\rho } }\right] \\\tag{6}\end{align*}

$W$ is the waiting time in the queue, $\text{T}_{\delta }$ it is the average transmission time for our proposed edge computing units and it is further defined in [30].

The Latency of the conventional network consists of few components, but in cloud architecture, it is a combination of the delay incurred due to irreducible propagation, transmission, routing, and the overall processing time. Sometimes few researchers suggested using software-defined networking switching delays as core to the main delay components [31]. Because delay increases as the number of users increase on particular geolocation for the cloud resource. The Following equation is formed for network latency at cloud without a third layer.

\begin{align*} L_{C}=&\left ({UE~upload~and~download~line }\right) \\&\ast \,\left ({minimum~distance~of~UE~to~the~nearest~AP }\right) \\&+\,\left ({AP~to~Cloud^{\prime }s~Upload~and~download~rate }\right) \\&\ast \, \left ({Distance~from~AP~to~Cloud }\right) \\&+\,(Observer~delay~in~IoT~node)\end{align*}

Similarly the total delay observed at edge computing is as follows.

\begin{align*} L_{e}=&\left ({UE~upload~and~download~line }\right) \\&\ast \,\left ({minimum~distance~of~UE~to~the~nearest~AP }\right) \\&+\,\left ({Observer~delay~in~IoT~node }\right) \\&+\,Control~plane~switching~latency\\&\times \, from~AP~ to~AP~with~in~edge\end{align*}

Aforementioned equation for latency in cloud and edge represented in Eq 6 and 7.

\begin{align*} L_{C}=&\left ({UD }\right)\ast \left ({{min}_{i=0}^{m}\left ({d_{UE-AP} }\right) }\right) \\&+\,\left ({{UD}_{C} }\right)\ast \left ({d_{AP-C} }\right)+(d_{IoT}) \tag{7}\\ \mathrm {L}_{\mathrm {e}}=&\left ({\mathrm {UD} }\right)\mathrm {\ast }\left ({{\mathrm {min}}_{\mathrm {i=0}}^{\mathrm {m}}\left ({\mathrm {d}_{\mathrm {UE-AP}} }\right) }\right)+\left ({\mathrm {d}_{\mathrm {IoT}} }\right)+\left ({\mathrm {d}_{\mathrm {s}} }\right)\tag{8}\end{align*}

## IV. Evaluation

We have created an environment to evaluate the efficiency of the proposed method. In this section, we have compared our method with another state-of-the art methods such as the FDDB, WIDER Face and Annotated facial Landmarks (AFLW). We have used FDDB data set which contains annotated 5171. Wider has more than half of them are used for testing categorized into 3 namely difficulty set, training (forty percent) set, and remaining validation subsets (10 %). Our test consists of placing devices and computational units within a building. Camera and thermos sensors placed on the Raspberry Pi computer placed on the device layer using wireless access points. The capture module and heat sensor to detect the temperature of residents are placed on these small computers. The edge layer is consists of server and database entities, all these servers are Linux based POSIX (Portable Operating System Interface) systems and SQLite is used as a database for faster retrieval of data and communication purposes.

### A. Training Data

WIDER FACE dataset is a face detection benchmark dataset, of which images are selected from the publicly available WIDER dataset. Since, MTCNN network uses face detection and alignment, however, we have used the only face detection, hence we have three types of annotations for training purposes. Negatives, we have used less than 0.3 as the ratio of the IoU regions to any ground truth face. The Positive region is more than 0.65 and landmark for facial localization. In the first stage of the MTCNN method is applied to crop WIDER FACES to arrange positive and negative and we have also used CelebA as landmark faces. In the seconds’ phase we have detected faces from the dataset of WIDER FACES and landmarked the faces from CelebA. In this article we detect a face, therefore we have utilized various data annotation technique for the training process firstly, Negatives, it is an area which is denoted by Intersection-over-Union (IoU) has less than 0.3 ratios to any ground-truth faces. The second one we have Positives, it is IoU ration is more than 0.65 to a ground truth face. Third, we call it a part of faces and has a ratio between 0.4 and 0.65 to a ground truth face. Finally, we have Landmark faces. The training data for each network as mentioned in the proposal is used as follows.

• P-Net Crop patches of WIDER face (To Derive Positive and negative, Part of face and face landmarks)
• R-Net (Use the out from the first stage to detect faces to mark positive, negative, part of the face and landmark from the CelebA dataset)
• O-Net (We have used this network to receive face data from this network. It is quite similar to the aforementioned network; however, we have utilized for face detection only)

**FIGURE 6.**
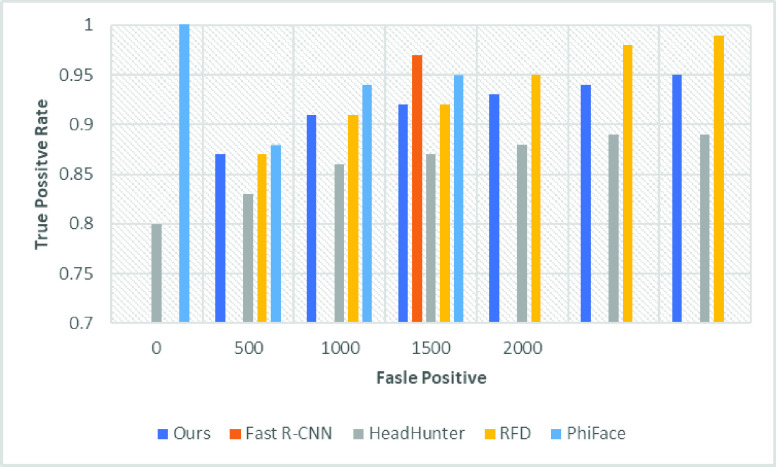
Evaluation of the proposed face detection on FDDB and WIDER Face.

**FIGURE 7.**
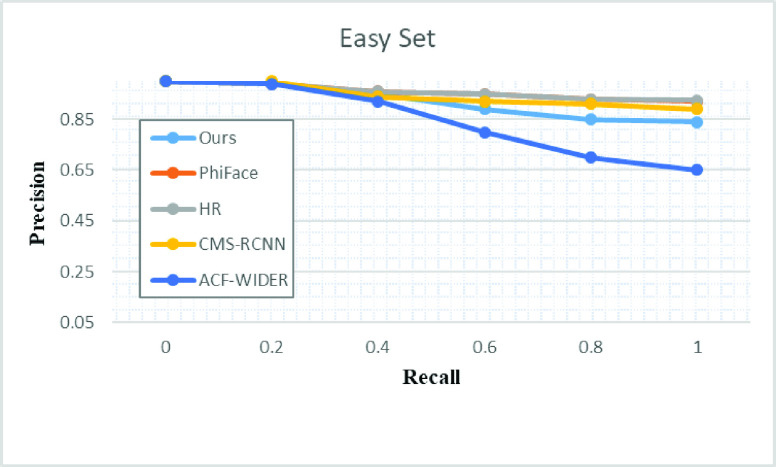
Evaluation of the proposed face detection on easy dataset FDDB and WIDER Face.

We have performed an evaluation of the proposed method of face detection and compared with the state-of-the-art [1]–[6] methods that have utilized FDDB datasets and WIDER FACE data set. Fig 6 and 7 and Table 2, shows that our proposed research work outclass all the earlier research proposals.

**TABLE 2:** Evaluation and Comparison of WIDERFACE With State of Arts Methodology

Average Precision/Recall Hardest	Ours	PhiFace	HR	CMS-RCNN	ACF-WIDER
	0. 607	0.810	0.806	0.624	0.273

For identifying face, we have collected data for different sized images and the time is taken by the processor to recognize the face. We have captured different sized images such as High definition images $1920\times 1080$ to the lowest sized image of $320\times 240$ image. The average time taken by the processor to process HD image is 100941 ms, this is result is calculated using 10 images. Similarly, when, the small-sized image processing time is 250ms. However, this processing time is directly proportional to the processing power the Raspberry Pi small computer.

We performed the experiments proposed system with respect to latency and detection. The Following settings were executed to measure the performance of the proposed system.

1. Moved the application server and database to the Edge layer
2. Hybrid settings: Equally distributed the load between Cloud and Edge computing
3. Moved computation entities to the Cloud

**FIGURE 8.**
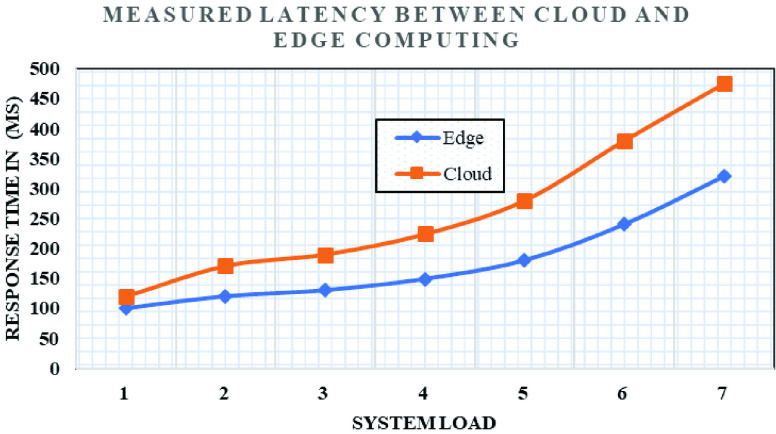
Latency of Cloud and Edge computing of Application and database server.

**FIGURE 9.**
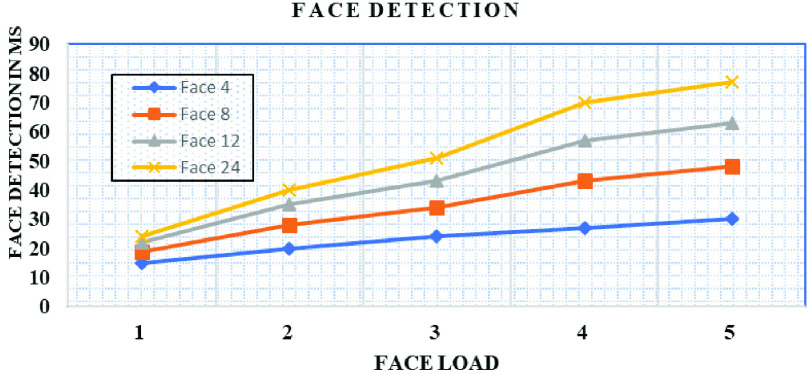
Face detection: average time taken by edge.

We hosted database server and application server at edge computing as shown in Fig 2. We ran several experiments to prove the efficiency of the proposed system against the settings of cloud-based services under the aforementioned experimental settings. We calculated latency for the aforementioned settings, and additionally, we also increased the device load on the current experiment settings. Fig 8, shows the latency of communication in Cloud and edge computing. Fig 9 shows an increase in face detection. It is the average face detection time in edge and cloud computing configuration. Figure 9 shows response time, measured separately for cloud and edge networks for the different numbers of face load when no bandwidth limitation on the edge and edge cloud architecture.

With these conditions, we have observed 45 ms response time with system load higher than 70% edge computing. For cloud-only architecture we have seen that the network latency making response time higher.

Figure 9 shows the bandwidth limitation of cloud computing on response time with variable load in the form number of faces. The limitation of bandwidth is between edge and cloud computing architecture. As the load increases, the detection time also increases under low bandwidth settings and whereas increased load on large bandwidth settings the average response time is an acceptable range.

**FIGURE 10.**
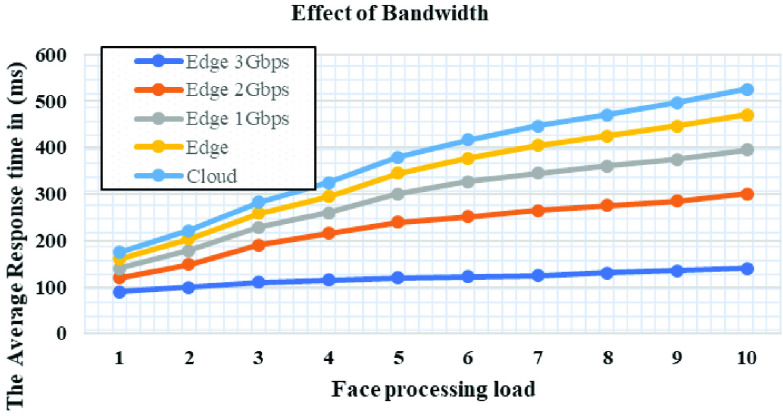
Average response time of the cloud and edge with different bandwidth setting.

**FIGURE 11.**
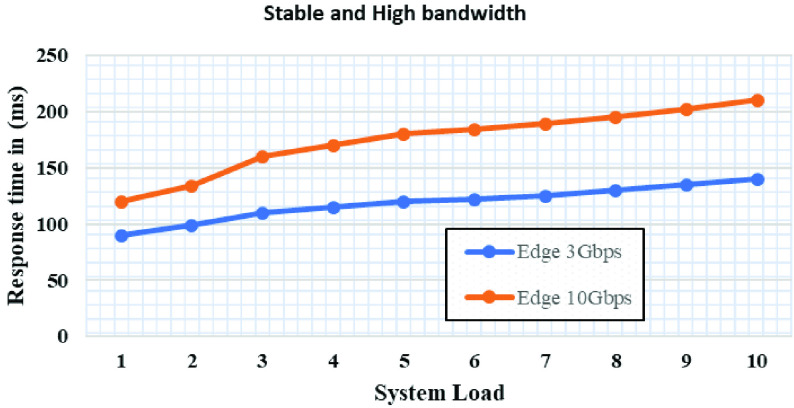
Average response time of the cloud and edge with stable and high bandwidth setting.

Figures 10 and 11 show the average response time of the cloud and edge system on different bandwidth bounds. We have two experiments, one is fixed front haul bandwidth that makes resource utilization. In the second condition set of an experiment in that a cloud-only with inter edge bandwidth. In the first experiments, edge computing only competed with unlimited bandwidth systems and hence it utilizes complete resources of edge only systems. In the second experiment, the cloud-only system performs better than the edge systems in inter edge bandwidth conditions, because when the edge unable to process new requests it passes the new requests to the neighboring edge using inter edge bandwidth. With these settings, response time will be increased because the bandwidth is utilized to satisfy application requirements. Additionally, propagation delay and queueing will also increase response time under extremely high load. As the load increases, the bandwidth will be no more left and hence the more queuing delay. As more response time delay causes application underperformance and it won’t handle more load of faces. Acceptable average response time for real-time image processing should not be more than 120ms. But if the bandwidth is increased, then propagation delay and queueing will not be noticed and the application will also have any trouble with the average response time. Figure 10 illustrates the required bandwidth to have constant fronthaul and inter edge bandwidth.

For testing purpose, we have extracted 10 faces per second for any given camera within the vicinity of the residential area. Our proposed method, if it receives 24 frames then the total number of frames is given.

\begin{align*} \text {Total number of frames}\,\, = \,\, &\text {Faces /seconds }^\ast 60 \text {minutes} \\ 10 ^\ast 60 ^\ast 60 \text {(minutes})\,\, = \,\, &1200~\text {frames}.\end{align*}

**FIGURE 12.**
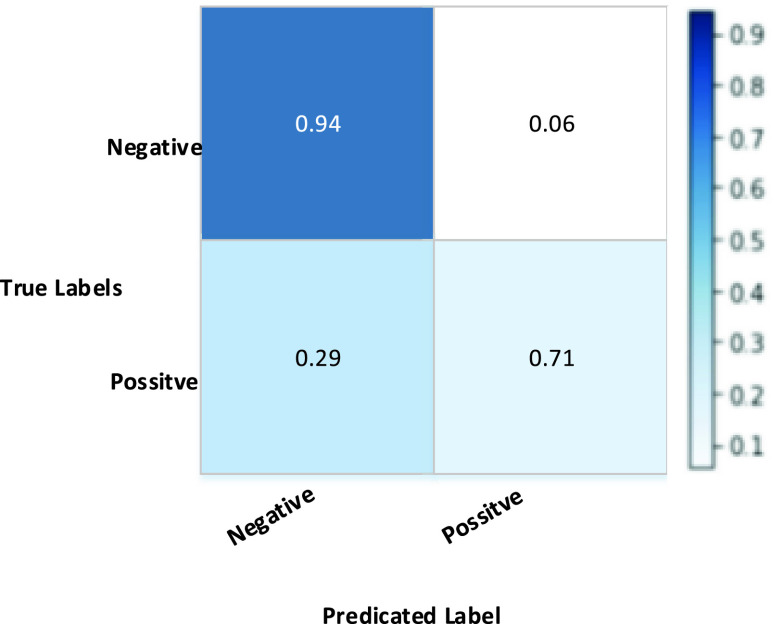
Normalized confusion matrix using our own dataset.

Therefore, it takes 10 hours to process one hour for 1200 frames. With the MTCNN, our proposed method has taken 1200 frame * 100 frames/ second = 1200/100 = 12 minutes. Fig 12, shows the confusion matrix obtained from the proposal using MCCNN network and obtained satisfactory results which coincides with the proposal.

## V. Conclusion

This research presented a decentralized IoT based solution for lockdown issues for the residential population to avoid flooding of people so as to follow guidelines. We have validated our proposal against the state-of-theart face detection and recognition methods under distributed and centralized architecture in terms of a load of devices and their work is done with respect to response time. For the face detection method, we have utilized a CNN based multi task cascaded framework. The performance shows that our research method performs better than the state-of-the-art proposals on various challenging datasets namely WIDER FACE (WIDER FACE dataset is a face detection benchmark dataset) and Face Detection Data Set and Benchmark. For decentralized conditions, we have compared the results of latency and load of devices on Cloud and Edge computing, on various centralized and distributed conditions of the application and database server. With respect to Edge computing we have achieved a better result on the decentralized load of face detection is achieved better results. In the future, we will study the essential features between face detection and its impact on the communication protocol and payload.

Funding

None.
